# Transgenic expression of omega-3 PUFA synthesis genes improves zebrafish survival during *Vibrio vulnificus* infection

**DOI:** 10.1186/s12929-015-0208-1

**Published:** 2015-11-17

**Authors:** Chih-Lun Cheng, Shin-Jie Huang, Chih-Lu Wu, Hong-Yi Gong, Chuian-Fu Ken, Shao-Yang Hu, Jen-Leih Wu

**Affiliations:** Institute of Cellular and Organismic Biology, Academia Sinica, Taipei, 115 Taiwan; Institute of Bioscience and Biotechnology, National Taiwan Ocean University, Keelung, 202 Taiwan; Institute of Fisheries Science, National Taiwan University, Taipei, 106 Taiwan; Department of Aquaculture, National Taiwan Ocean University, Keelung, 202 Taiwan; Institute of Biotechnology, National Changhua University of Education, Changhua, 500 Taiwan; Department of Biological Science and Technology, National Pingtung University of Science and Technology, Pingtung, 912 Taiwan

**Keywords:** Omega-3 PUFAs, Anti-inflammation, Anti-bacteria, *Vibrio vulnificus*, Transgenesis

## Abstract

**Background:**

Highly desaturated n-3 polyunsaturated fatty acids (PUFAs), such as eicosapentaenoic acid (EPA) and docosahexaenoic acid (DHA), are synthesized by desaturases and elongase. They exert hepatoprotective effects to prevent alcoholic fatty liver syndrome or cholestatic liver injury. However, it is unclear how n-3 PUFAs improve immune function in liver. *Vibrio vulnificus*, a gram-negative bacterial pathogen, causes high mortality of aquaculture fishes upon infection. Humans can become infected with *V. vulnificus* through open wounds or by eating raw seafood, and such infections may result in systemic septicemia. Moreover, patients with liver diseases are vulnerable to infection, and are more likely than healthy persons to present with liver inflammation following infection. This study quantified n-3 PUFAs and their anti-bacterial effects in Fadsd6 and Elvol5a transgenic zebrafish.

**Results:**

Two transgenic zebrafish strains with strong liver specific expression of Fadsd6 and Elvol5a (driven by the zebrafish *Fabp10* promoter) were established using the Tol2 system. Synthesis of n-3 PUFAs in these strains were increased by 2.5-fold as compared to wild type (Wt) fish. The survival rate in 24 h following challenge with *V. vulnificus* was 20 % in Wt, but 70 % in the transgenic strains. In addition, the bacteria counts in transgenic fish strains were significantly decreased. The expression levels of pro-inflammatory genes, such as *TNF-α*, *IL-1β*, and *NF-κB*, were suppressed between 9 and 12 h after challenge. This study confirms the anti-bacterial function of n-3 PUFAs in a transgenic zebrafish model.

**Conclusions:**

Fadsd6 and Elvol5a transgenic zebrafish are more resistant to *V. vulnificus* infection, and enhance survival by diminishing the attendant inflammatory response.

**Electronic supplementary material:**

The online version of this article (doi:10.1186/s12929-015-0208-1) contains supplementary material, which is available to authorized users.

## Background

Docosahexaenoic acid (DHA, 22: 6 n-3) and eicosapentaenoic acid (EPA, 20:5 n-3) are formed through desaturation and elongation of α-linolenic acid (ALA, 18: 3 n-3), as catalyzed by pivotal desaturases and elongase [1, 2]. Omega-3 polyunsaturated fatty acids (n-3 PUFAs), such as DHA and EPA, are abundant in marine organisms. Salmon, tuna, and mackerel can efficiently synthesize EPA and DHA after feeding on marine algae and phytoplankton that contain large amounts of ALA. However, most vertebrates, including humans, cannot synthesize high levels of long chain n-3 PUFAs because the essential desaturases in these species are not sufficiently efficient [3]. Fish oils are thus important and abundant sources of long chain n-3 PUFAs for humans [2, 4, 5]. However, over-consumption and heavy metal contamination of marine fishes are critical problems for their use in the human diet [6–8]. Advances in aquaculture techniques and transgenesis have enabled the farming of fish that synthesize high levels of n-3 PUFAs, and thus farmed fish unexposed to metal pollution can substitute for marine fishes as sources of fish oils.

Various n-3 PUFAs have been reported to exert beneficial effects, such as protection against liver diseases, regulation of cholesterol, and reduction of blood pressure, which prevents cardiovascular diseases (CVDs) [9, 10]. On the other hand, some studies have indicated that n-3 PUFAs exert anti-inflammatory effects by regulating the expression of peroxisome proliferator activated receptors (PPARs) and nuclear factor kappa B (NF-κB) [11–13]. The inflammatory response is triggered by activation of NF-κB, which induces the expression of pro-inflammatory cytokines, adhesion molecules, chemokines, growth factors, and inducible enzymes, such as cyclooxygenase 2 (COX-2) and nitric oxide synthase (NOS) [14]. In an earlier study, COX was shown to be able to convert arachidonic acid (AA, 20:4 n-6) into prostaglandin (PG)-H_2_ to enhance inflammation. In contrast, the other substrate for COX is EPA, which is not only an inhibitor of AA metabolism, but also an alternative substrate for COX-mediated synthesis of PGH_3_, an anti-inflammatory cytokine. Furthermore, other inflammatory mediators, such as tumor necrosis factor-α (TNF-α) and interleukin-1β (IL-1β), are inhibited by EPA [8]. Hence, n-3 PUFAs improve many chronic syndromes by suppressing inflammation [9].

EPA and DHA also exhibit significant anti-bacterial effects against *Propionibacterium acnes* and *Staphylococcus aureus* [15]. In 2005, Li et al. demonstrated that both EPA and DHA down-regulate lipopolysaccharide (LPS) -induction inflammation in human kidney-2 (HK-2) cells [16]. These findings indicate that n-3 PUFAs are efficient at preventing inflammation induced by bacterial infection.

*Vibrio vulnificus* (*V. vulnificus*) is a gram-negative bacterium which causes infectious disease and striking mortality, mostly due to septicemia [17]. Infection in humans is caused by wound exposure or consumption of raw sea food. Rapid progression to septicemia following *V. vulnificus* infection is associated with high mortality [18]. Moreover, patients with liver diseases are particularly vulnerable to infection, and are more likely than healthy persons to present with inflammation upon infection [19]. On the other hand, vibriosis has resulted in serious economic losses in aquaculture in Japan and Europe [20]. *V. vulnificus* induces the host immune response through TLRs and their downstream genes, NF-κB, which translocates to the nucleus and leads to inflammation [21, 22].

Most fresh water fish lack the n-3 PUFAs and can’t protect from bacterial infection. We try to increase the n-3 PUFAs by transgenic fish. Because the liver is the major organ for lipid metabolism, it is also a target of *V. vulnificus* [23]. According to the n-3 PUFAs biosynthesis pathway, fatty acid desaturase-Δ6 (Fadsd6) is the rate limited step and elongase (Elvol5a) is also important [3, 24]. In this study, we demonstrated that liver-specific overexpression of *Fadsd6* and *Elvol5a* in transgenic zebrafish exerts protection from *V. vulnificus* infection. Our findings suggest that liver-specific expression of Fadsd6 or Elvol5a enhances the bio-synthesis of EPA and DHA in transgenic zebrafish, and this is sufficient to increase the survival rate in response to *V. vulnificus* challenge.

## Methods

### Zebrafish maintenance

Wild-type (AB) zebrafish (*Danio rerio*) was maintained under standard conditions (water-flow tanks at 28.5 °C with a 12 h light/12 h dark cycle). Zebrafish was fed on a commercial diet twice a day. The embryos were collected through natural matching and cultured in a 28.5 °C incubator. All conditions for maintaining zebrafish were as previously described (Westerfield, M., 2007) [25].

Research was conducted in compliance with the principles stated in the Guide for the Care and Use of Laboratory Animals, National Research Council, 1996. All animal experiments in this study were approved by the Academia Sinica Institutional Animal Care & Utilization Committee (AS IACUC).

### Construction of transgenic fish

Plasmids contained the *Fabp10* promoter, which was used to drive a tetracycline-controlled transactivator (tTA) and either the TcFP13 (GFP) or TcFP11 (RFP) reporter genes. The *Fadsd6* and *Elvol5a* genes of Atlantic salmon (*Salmo salar*) were individually flanked by the Tol2 transposon element, and placed downstream of the tetracycline responsive element. This liver-specific gene expression system was modified from that described in our previous publication [26].

Transposase messenger RNA (mRNA) was co-injected with vectors into embryos (at the one-cell stage), and excision efficiency assays were performed as described [27]. Injected fish were cultured to sexual maturity and outcrossed to identify germ line transgenic fish.

### Zebrafish RNA extraction and quantitative RT-PCR

RNA from zebrafish liver tissue was extracted with TRIzol reagent (Invitrogen) and reverse transcribed to cDNA using the High Capacity cDNA Reverse Transcription Kit (Applied Biosystems). Quantitative real time-PCR was performed using a LightCycler480 system (Monocolor hydrolysis UPL-probe, Roche Applied Science). The Q-PCR synthesis mix reagents included 5 μL Master Buffer (Roche Applied Science), 2 μL each primer (2 μM), 0.1 μL probe (the numbers in Table 1 correspond to each primer pair), 2.5 μL cDNA (80 mg/mL, diluted 100 times), and 0.4 μL ultra-pure water. The sequences of the primers used are listed in Table 1. For analysis of gene expression, we determined the relative expression value (2^-ΔΔC^_T_) for each gene by subtracting the ΔC_T_ value of the control sample from that of the infected sample (i.e. ΔΔC_T_). Values are presented as relative fold expression levels with the standard error; results from at least three independent experiments were averaged.

**Table 1:** Primer list of RT-PCR and quantitative RT-PCR

Primer	Gene name	Sequence	Universal probe NO.
EF-1α	Elongation factor-1alpha	F: cctctttctgttacctggcaaa	# 73
		R: cttttcctttcccatgattga	
TNF-α	Tumor necrosis factor-alpha	F: aggcaatttcacttccaagg	# 158
		R: aggtctttgattcagagttgtatcc	
COX-2a	Cyclooxygennase-2a	F: agccctactcatcctttgagg	# 161
		R: tcaaccttgtctacgtgaccata	
IL-1β	Interleukin-1beta	F: acgtctccacatctcgtactca	# 12
		R: tcgaaggtgtttatggagctg	
IL-15	Interleukin-15	F: aggctcaggagaagactcacc	# 9
		R: ggatgtcgtgctgagcaat	
Lysozyme	Lysozyme	F: gggattctccattggcaac	# 48
		R: ctcggtgggtcttaaacctg	
NF-κB 1	Nuclear factor kappa B1	F: gcatctgcatctccgaca	# 109
		R: tgcccaaattagggaaactg	
Fadsd2	Fatty acid desaturase 2	F: tcaggaccggcagaaaaa	#41
		R: aacagcggctgcgtttta	
Elovl2	Elongase 2	F: tggacagcctatttggagaaa	#66
		R: gcaaaaatgttggtgtgtagga	
Elovl5	Elongase 5	F: ccaaatctcttacatggtcacg	#15
		R: tcccgaacgtttcttataggtc	
RT-Fadsd6 (V5-tag)	Fatty acid desaturase delta6	F: atggggggcggaggccagcag	-
		R: gaatcgagaccgaggagaggg	
RT-Elvol5a (V5-tag)	Elongase	F: atggagacttttaattataaac	-
		R: gaatcgagaccgaggagaggg	
RT- β-actin	Beta-actin	F: cacggcatcattaccaactg	-
		R: gtacggccagaagcgtagag	

### Western blots and antibodies

To examine expression of proteins in transgenic fish, total proteins were extracted from zebrafish tissue (approximately 50 mg) and added to 500 μL 2-D rehydration sample buffer with 5 μL ready prep TBP reducing agent (Ready-Prep Protein Extraction Kit, Bio-Rad). After centrifugation at top speed for 20 min at 18 °C, 300 μL supernatants were collected and transferred to new 1.5 mL microcentrifuge tubes, and kept at -80 °C for long-term storage. Protein concentrations were determined using a Protein Assay Kit (595 nm, Bio-Rad). For Western blot, protein samples (100 mg) were separated using the NuPAGE Bis-Tris Mini Gel Kit (Invitrogen) and transferred to a 0.45 nm PVDF membrane (Millipore, Bedford) using a trans-blot SD cell (Bio-Rad). Membranes were blocked with 5 % milk (Anker) for 1 h, and then rinsed three times with PBS (Bioman) for 5 min with shaking; next, membranes were incubated with primary Anti-V5 antibody (1:1000 dilution; Invitrogen) overnight at 4 °C. Samples were incubated with β-actin antibody (1:5000 dilution; Invitrogen) as an internal control; after washing to remove excess antibody, samples were incubated with secondary antibody (anti-mouse; Millipore) for 1 h at room temperature with shaking, and then washed as described above. Membranes were exposed to Immobile Western Chemiluminescent HRP Substrate (Millipore), and proteins were then detected using an Imaging System (UVP).

### Fatty acid extraction and analysis

For fatty acid analysis of transgenic lines and wild-type zebrafish, total lipids were extracted with organic solvent (chloroform: methanol 2:1, containing BHA 0.05 mg, Sigma), using a previously described method (Folch et al. 1957) [28]. Crude lipids were saponified through incubation for 20 min (reflux at 90 °C) with 1 mL of KOH (50 %, Merck), 15 mL of alcohol (90 %, Merck), and boiling stones. After being allowed to cool, the hydrophilic samples were purified and washed with 30 mL water and then 40 mL ethyl ether (this wash step was repeated three times). Pure fatty acids in the organic layer were reduced through the addition of 50 mL of ethyl ether, 2-3 drops of methyl orange, and 10 mL of 2 N HCl (Merck); the organic layer was then shaken lightly for 1 min. The organic layer (approximately 50 mL), which contains hydrophobic samples, was washed with water (50 mL) 4-5 times until the pH value became neutral; the layer was then evaporated with a vacuo concentrator (Eyela). During the methyl esterification steps, the sample (approximately 100 mg) was incubated with 5 mL of BF_3_-MeOH 7 % (Boron trifluoride-methanol solution, 14 %, Sigma) and boiling stones for 40 min (reflux at 90 °C). Subsequently, 5 mL hexane (Sigma) was added, and the sample was incubated for an additional 1 min. Fatty acid methyl esters (approximately 5 mL) were dried and diluted with highly pure hexane (100 mg/mL). The samples were analyzed using an Agilent 5975C Series GC-MSD (Agilent). The Agilent column was 30 mm X 0.25 mm, with a film thickness of 0.25 mm (Crawford Scientific, Strathaven); conditions were as previously described (Abu, 2009) [29].

### Bacterial culture and challenge

*V. vulnificus* was cultured on BHI (Brain heart infusion powder, BD Difco™) agar plates containing 1.5 % w/v sodium chloride (Merck) in a 30 °C incubator for 16 h. A single colony of *V. vulnificus* was subsequently incubated in 300 mL BHI (NaCl 1.5 %) media in a 30 °C incubator on a shaker operating at 200 rpm for a further 16 h. Subsequently, 30 μl of bacteria were cultured with 300 μL of BHI (NaCl 1.5 % w/v) media in a 30 °C incubator on a shaker operating at 200 rpm to obtain the appropriate concentration (10^7^ CFU/mL).

Wild-type and transgenic zebrafish were challenged by intraperitoneal injection of 10 μL *V. vulnificus* (10^4^ CFU/mL, diluted with PBS). Five fish were sacrificed at 0, 1, 3, 6, 9, 12, 24, and 48 h after challenge for each group.

Liver samples were homogenized in BHI (NaCl 1.5 %, 1 mg/20 μL) media, and the supernatants were serially diluted in BHI (NaCl 1.5 %, 1 mg/20 μL) media. Ten microliters of each dilution were spotted onto a TCBS (Thiosulfate-citrate-bile salts-sucrose, powder, BD Difco™) plate. Plates were incubated in a 30 °C incubator for 16 h, and *V. vulnificus* colonies were then counted.

### Histological studies and TUNEL assay

Liver tissue was fixed with 10 % neutral formalin (Sigma) and embedded in paraffin. Sections with a thickness of 5 mm were prepared, and then stained with hematoxylin and eosin (H&E, Thermo) for histological examination. DNA damage resulting from challenge with *V. vulnificus* was detected by subjecting paraffin-embedded sections to the TUNEL assay, using the *In Situ* Cell Death Detection kit, POD (Roche Applied Science).

### Statistical analysis

The expression values of all the data are presented as the average ± standard deviation (SD). Statistical analysis was performed using Student’s *t*-test, and significance was set at *P < 0.05, **P < 0.01.

## Results

### Fadsd6 and Elvol5a are expressed in transgenic zebrafish liver

**Fig. 1:**
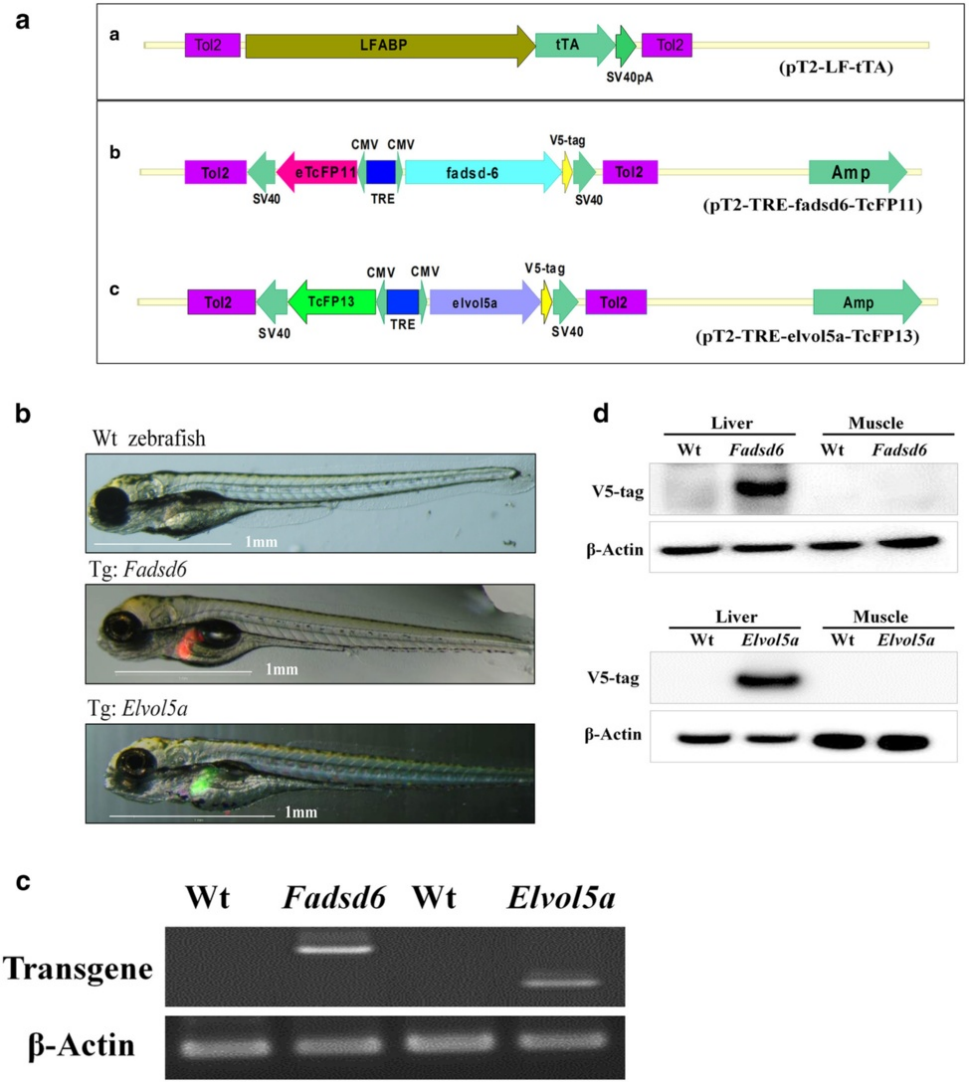
Transgenic zebrafish specifically express Fadsd6 and Elvol5a in liver. (**a**) Schematics of the liver-specific activator plasmid pT2-LF-Tta (containing an activator (tTA) driven by the zebrafish *fabp10* promoter) and two tetracycline-responsive plasmids (pT2-TRE-fadsd6-TCFP11 and pT2-TRE-elvol5a-TCFP13). All expression cassettes are flanked by the Tol2 transposon. (**b**) Fluorescence microscopy images of F3 Fadsd6 (red) and Elvol5a (green) transgenic zebrafish larvae, taken at five days (5D) after hatching. (**c**) RT-PCR was used to detect exogenous gene expression of Fadsd6 and Elvol5a in adult transgenic and Wt zebrafish. (**d**) Western blots were performed to detect fusion of the V5-tag to Fadsd6 and Elvol5a in transgenic and Wt zebrafish. Mouse β-actin was used as a positive control

Two transgenic zebrafish lines with strong liver-specific expression of Fadsd6 and Elvol5a were generated. Each plasmid contained the *Fabp10* promoter, which was used to drive a tetracycline-controlled transactivator (tTA) and either the TcFP13 (GFP) or TcFP11 (RFP) reporter genes. The *Fadsd6* and *Elvol5a* genes of Atlantic salmon (*Salmo salar*) were flanked by the Tol2 transposon element, and placed downstream of the tetracycline responsive element (Fig. 1 (a)).

Either green or red fluorescence was observed in the liver of stably transgenic larvae expressing Fadsd6 or Elvol5a, respectively (Fig. 1 (b)). Ectopic gene expression of Fadsd6 or Elvol5a was detected in transgenic fish liver by the amplification of RT-PCR (Fig. 1 (c)). Western blots were performed to detect V5-tagged Fadsd6 or Elvol5a protein in liver tissue of transgenic fish, with β-actin as an internal control (Fig. 1 (d)). In summary, the transgenic zebrafish lines specifically overe-xpressed Fadsd6 and Elvol5a in liver.

### Synthesis of n-3 PUFAs was 2.5 - fold greater in Fadsd6 and Elvol5a transgenic zebrafish than in Wt

**Fig. 2:**
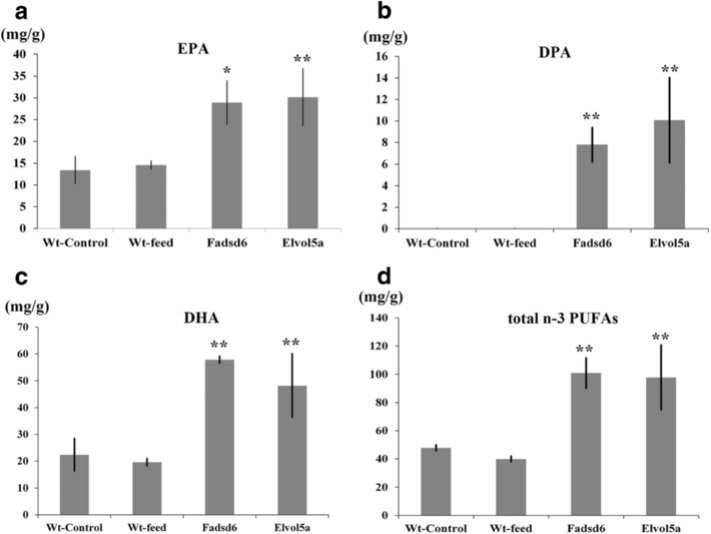
Polyunsaturated fatty acids in transgenic and Wt zebrafish were measured through gas chromatography-mass spectrometry (GC-MS). (**a**) Eicosapentaenoic acid (EPA), (**b**) docosapentaenoic acid (DPA), (**c**) docosahexenoic acid (DHA), and (**d**) total n-3 PUFAs were analyzed. Control groups of Wt fish were fed on artemia, while other groups were fed on a commercial diet. The values are milligrams of fatty acid per gram of whole fish body, and are shown as means ± SEM (*n* = 5). Statistically significant differences between Wt and transgenic fish were determined using *T*-TEST (*p < 0.05, **p < 0.01)

Total lipid content was not significantly different between transgenic fish and Wt fish (Additional file 1: Table S1). EPA content was approximately 2.0-fold higher in both Fadsd6 and Elvol5a transgenic fish than in Wt (Fig. 2 (a), Additional file 1: Table S1), while DPA content was 9.4-fold and 12.1-fold higher in Fadsd6 and Elvol5a transgenic fish than in Wt, respectively (Fig. 2 (b), Additional file 1: Table S1). DHA content was 2.9-fold and 2.5-fold higher in Fadsd6 and Elvol5a transgenic fish than in Wt, respectively (Fig. 2 (c), Additional file 1: Table S1). As expected, total n-3 PUFAs content was 2.5-fold higher in Fadsd6 and Elvol5a transgenic fish than in Wt, respectively (Fig. 2 (d), Additional file 1: Table S1). No significant difference was observed between Wt fish fed on a commercial diet or artemia (Additional file 1: Table S1). These results indicate that n-3 PUFAs synthesis is enhanced in transgenic fish over-expressing either Fadsd6 or Elvol5a.

### Transgenic fish exhibit high survival rates during V. vulnificus challenge

We proceeded to examine whether high n-3 PUFA contents suppress bacterial infection. The bodies of transgenic and Wt fish were IP-injected with 10 μL *V. vulnificus* in BHI (10^6^ CFU/ mL, NaCl 1.5 %, diluted to 10 % with 1X PBS). One group of Wt fish was injected with 10 μL 1X PBS as a negative control. Each group contained at least 30 fish for one test. Fish mortality was determined at 0, 1, 3, 6, 9, 12, 18, 24, and 48 h after challenge.

**Fig. 3:**
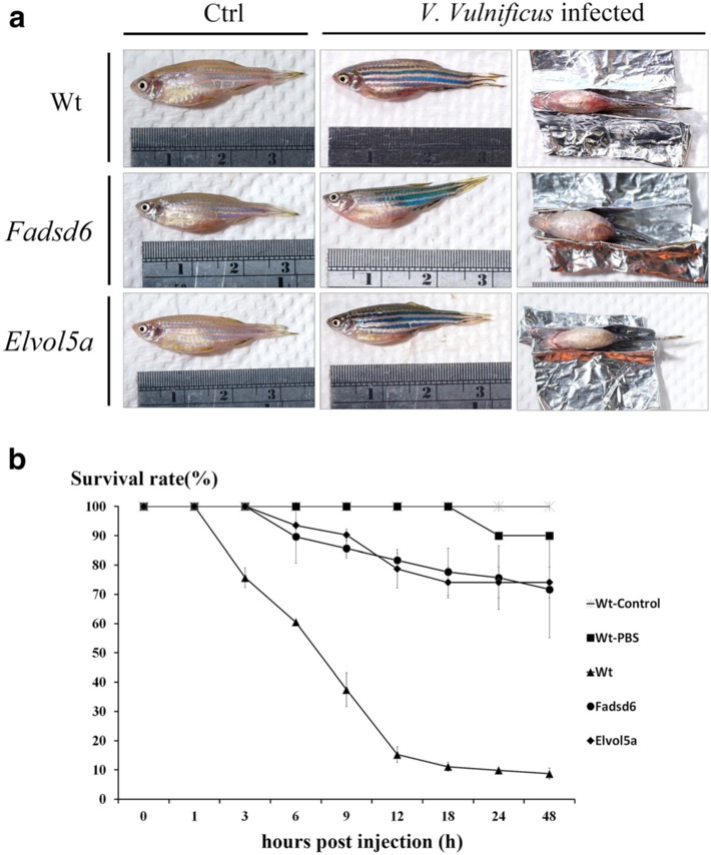
Survival rates of zebrafish after infection with *V. vulnificus* for 48 h (**a**) Photographs of Wt zebrafish control (left) and Wt fish challenged with *V. vulnificus* (middle and right) at 9 h after IP injection revealed bleeding at the ventral sides of the infected fish body. (**b**) Survival rates of Wt and transgenic fish injected with *V. vulnificus* (Wt-control was not infected and Wt-PBS was injected with PBS). Survival rates were determined at the indicated times after infection. Each experimental group contained 30 adult zebrafish

Infected fish lost balance in 3-6 h. The ventral side of infected Wt fish was observed to be bleeding at 9 h and with some speckle of blood on its body after challenge with *V. vulnificus.* However, both two transgenic fish showed slight bleeding. The skin looks quite smooth as normal fish (Fig. 3 (a)). The survival rate of infected Wt fish decreased dramatically between 9-12 h, and dropped to less than 10 % by 24 h after infection. However, the survival rates of Fadsd6 and Elvol5a transgenic fish remained at up to 70 % at 24 h after challenge with *V. vulnificus* (Fig. 3 (b)). Taken together, these results indicate that both transgenic lines with higher contents of n-3 PUFAs were more resistant than Wt zebrafish to *V. vulnificus* challenge.

### Pathogen-induced liver damage was suppressed in transgenic fish

**Fig. 4:**
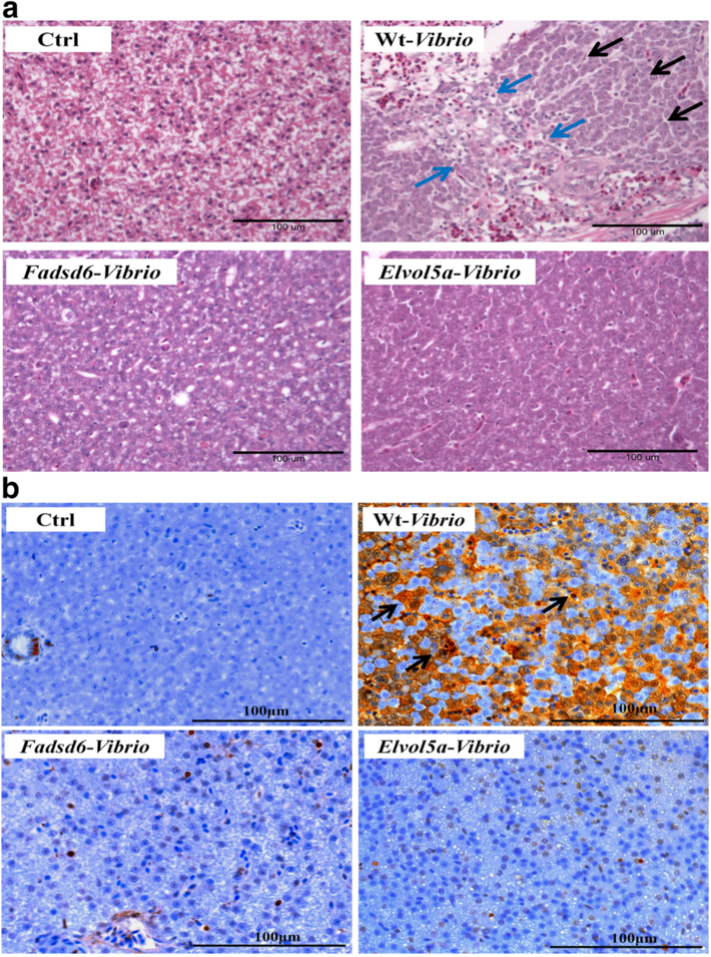
Histology of liver sections after challenge with *V. vulnificus* (**a**) H&E (Hematoxylin and eosin) staining revealed hepatocyte damage (black arrows) and lymphocyte infiltration (blue arrow) in Wt fish after challenge with *V. vulnificus.* (**b**) TUNEL-assay was performed to confirm cell death (brown signals, indicated by black arrows) in Wt fish after challenge with *V. vulnificus.* Scale bars = 100 μm

Examination of liver histopathological sections revealed that serious liver damage occurred in infected Wt fish. Swollen nuclei, ruptured cells (with an increased ratio of nuclei to cytoplasm), lymphocyte infiltration, and extensive monocyte recruitment were observed within the livers of Wt fish. However, Fadsd6 and Elvol5a transgenic fish did not exhibit substantial damage after infection (Fig. 4 (a)). In addition, TUNEL-assay was performed to identify hepatocyte damage caused by *V. vulnificus.* While cell damage was observed in Wt fish, it was less apparent in transgenic fish (Fig. 4 (b)). The signals of TUNEL assay were quantitated (Additional file 2: Figure S2). In summary, these results indicate that liver damage caused by *V. vulnificus* infection was attenuated in both transgenic lines.

### Bacterial growth was inhibited in transgenic fish after V. vulnificus challenge

**Fig. 5:**
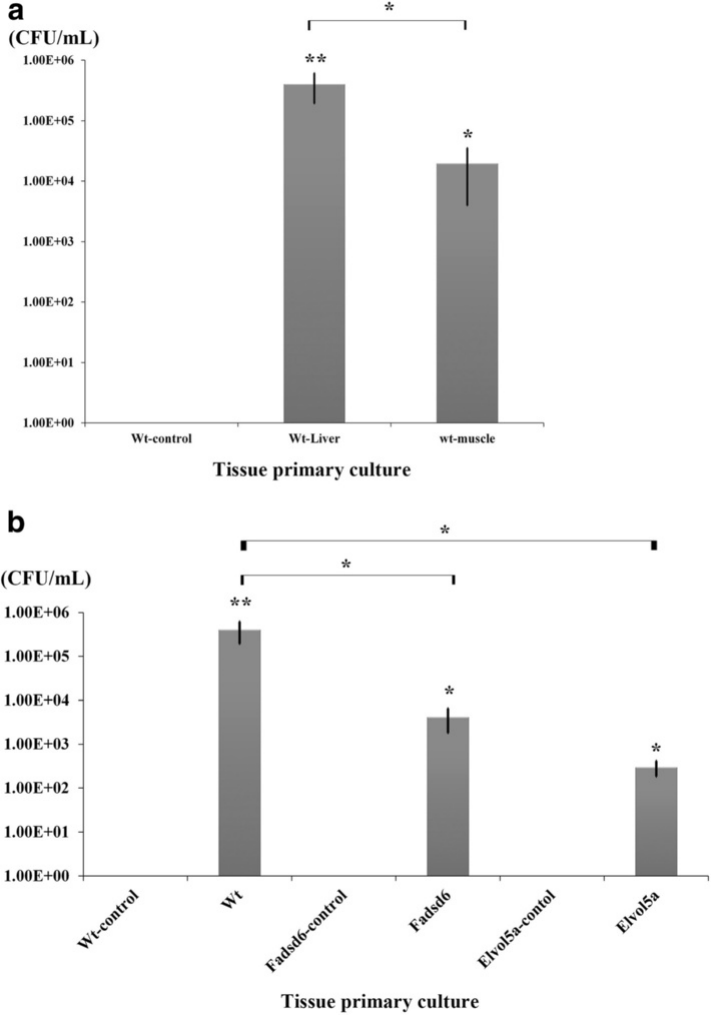
Bacterial contents were determined in zebrafish after challenge with *V. vulnificus*. Tissues were collected and cultured on TCBS agar plates. (**a**) Bacterial amounts (CFU/mL) were determined in Wt liver or muscle and (**b**) in liver of Fadsd6 or Elvol5a transgenic fish. Values are presented as means ± SEM. Significance was determined by *T*-TEST (*P < 0.05, **P < 0.01)

We next examined whether bacterial growth after *V. vulnificus* challenge is affected by over-expression of Fadsd6 or Elvol5a. Bacteria were re-cultured on a TCBS agar-plate from tissue homogenates. At 9 h post-challenge, the bacterial contents of both Wt liver and muscle homogenates were significantly higher than those in non-infected Wt fish. CFU count was 20.64-fold higher in Wt liver than in muscle (Fig. 5 (a)). Moreover, CFU counts were 97.4 and 1353.3-fold higher in Wt liver than in Fadsd6 and Elvol5a transgenic fish, respectively (Fig. 5 (b)). Altogether, these findings indicate that the high contents of n-3 PUFAs in both Fadsd6 and Elvol5a transgenic fish can inhibit bacterial growth after *V. vulnificus* challenge.

### Inflammatory gene expression was down-regulated in transgenic fish

**Fig. 6:**
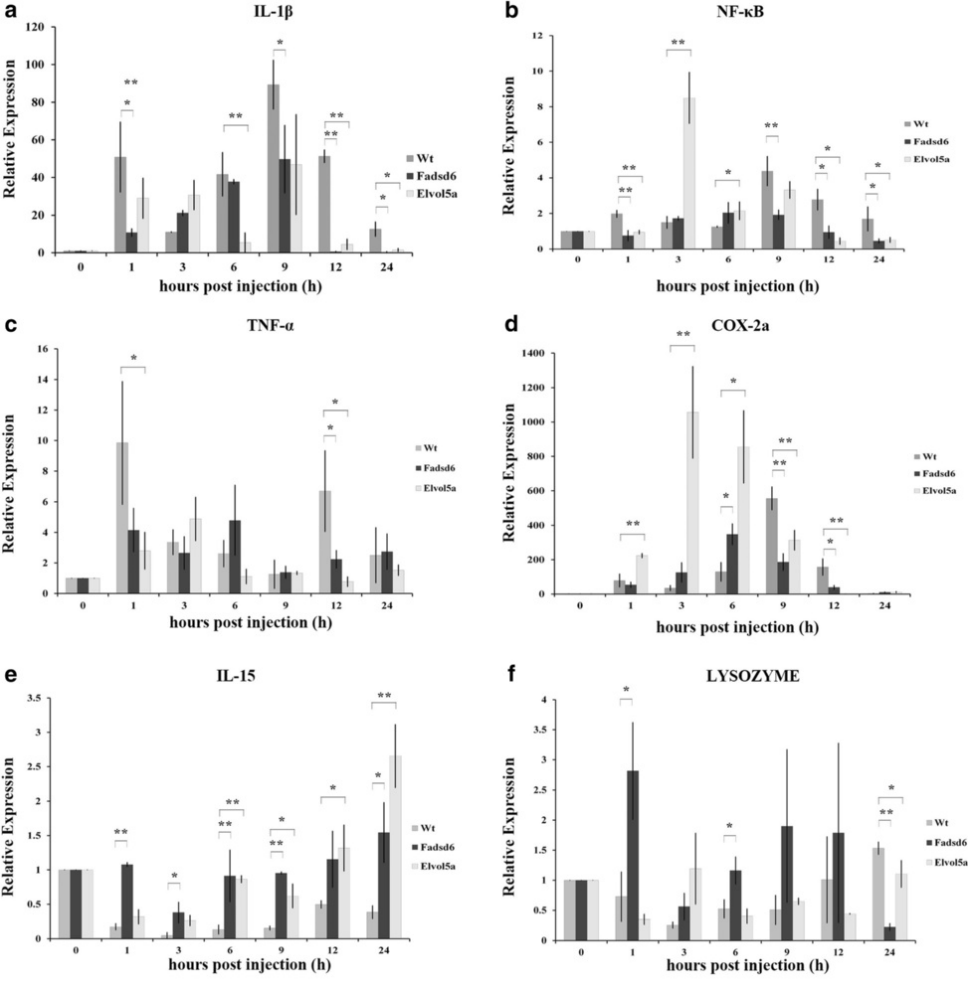
Inflammatory gene expression in transgenic and Wt zebrafish at the indicated times after challenge with *V. vulnificus*. Expression was determined by real-time qPCR. (**a**) IL-1β, (**b**) NF-κB, (**c**) Tnf-α, (**d**) Cox-2a, (**e**) IL-15, and (**f**) Lysozyme. Values are presented as means ± SEM. Significance was determined by *T*-TEST (*P < 0.05, **P < 0.01)

Finally, we examined the expression levels of inflammation-associated genes in the livers of Wt and transgenic zebrafish after challenge with *V. vulnificus*. Expression levels of pro-inflammatory genes were strongly increased in Wt fish (Fig. 6). Expression of IL-1β at 9 h post-injection in Wt fish was 1.8-fold higher than in Fadsd6 transgenic fish, whereas the expression levels at 12 h were 86.8-fold and 11.4-fold higher than in Fadsd6 and Elvol5a transgenic fish, respectively (Fig. 6 (a)). For NF-κB, expression in Wt at 9 h post-injection was 2.3-fold higher than in Fadsd6 transgenic fish, and expression in Wt at 12 h post-injection was 2.9-fold and 6.2-fold higher than in Fadsd6 and Elvol5a transgenic fish, respectively (Fig. 6 (b)). Expression of TNF-α at 12 h post-injection in Wt was 3.0-fold and 8.7-fold higher than in Fadsd6 and Elvol5a transgenic fish, respectively (Fig. 6 (c)). For Cox-2a, expression in Wt at 9 h post-injection was 3.0-fold and 1.8-fold higher than in Fadsd6 and Elvol5a transgenic fish, respectively, while, expression at 12 h post-injection in Wt was 4.1-fold and 13.7-fold higher than in Fadsd6 and Elvol5a transgenic fish, respectively. (Fig. 6 (d)). For IL-15 and lysozyme, expression levels were suppressed in Wt fish after challenge with *V. vulnificus.* Expression levels of IL-15 in Fadsd6 transgenic fish were 5.9-fold and 2.3-fold higher than in Wt at 9 and 12 h post-injection, respectively, and IL-15 levels in Elvol5a transgenic fish were 3.9-fold and 2.6-fold higher than in Wt at 9 and 12 h post-injection, respectively (Fig. 6 (e)). At 1 and 6 h, expression levels of lysozyme in Fadsd6 transgenic fish were 3.9-fold and 2.2-fold higher than in Wt, respectively (Fig. 6 (f)).

Overall, these results indicate that the expression levels of pro-inflammatory genes are decreased in both Fadsd6 and Elvol5a transgenic fish, and this may suppress the inflammatory response to challenge with *V. vulnificus* within a 24 h window.

## Discussion

Long-chain n-3 PUFAs, such as EPA and DHA, are well-known to have many beneficial effects on biological functions. However, total fat content and the ratio of n-3 to n-6 PUFAs in food have decreased significantly since the Industrial Revolution [30]. Moreover, the efficiencies of desaturases and elongase in mammals are too low to generate high n-3 PUFA contents. Therefore, in 2005, 2007, and 2008, Alimuddin et al. established transgenic zebrafish that expressed Fadsd5/6 and Elvol5a under the control of the β-actin promoter [3, 24, 31]. Transgenic zebrafish described in these earlier studies synthesized about 1.3-fold more EPA, DPA, and DHA than Wt. In our findings, the expression levels of both the endogenous desaturase (Fadsd2) and elongase (Elovl2) genes were significantly higher in Wt liver than in muscle (Additional file 3: Figure S1). Taken together, the evidences show these desaturase and elongase genes are tissue specific which enhances the efficiency of long chain fatty acid synthesis. A more recent study described the establishment of transgenic zebrafish specifically expressing Fadsd5 and Fadsd6 in the muscle, through the use of the myosin light chain (mlc3) promoter [32]. EPA content in Fadsd5 and Fadsd6 transgenic fish were 2.0 and 1.9-fold higher than in Wt, respectively, but there was no significant difference in DHA and DPA contents. Indeed, this aforementioned study demonstrated that tissue-specific expression of desaturases and elongase is sufficient to enhance n-3 PUFA biosynthesis in transgenic fish. Transgenic fish expressing Fadsd6 and Elvol5a specifically in the liver under the control of the *Fabp10* promoter was created for the following reasons: (1) the liver is the major organ for lipid metabolism; (2) Fadsd6 has been shown to be rate limiting for the conversion of ALA to EPA in the lipid biosynthesis pathway; and (3) Elvol5a improves the production of DHA and DPA in the n-3 PUFA biosynthesis pathway [33, 34]. When body weights are similar, Fadsd6 and Elvol5a transgenic fish synthesized 2.5-fold more n-3 PUFAs than Wt. The contents of n-3 PUFAs were also higher than those in transgenic fish expressing the same genes under the control of β-actin or mlc3 promoter. Liver specific-expression of these genes is thus more efficient than global expression at producing n-3 PUFAs.

A separate study showed that increasing n-3 PUFA content reduced the generation of eicosanoids from AA and promoted the generation of PGH_3_ [35]. These n-3 PUFAs compete with the enzymes that convert AA into PG, thereby decreasing the production of pro-inflammatory prostaglandins. Furthermore, *in vitro* studies have indicated that EPA suppresses the growth of *Staphylococcus aureus* and *Propionibacterium acnes* [15, 36]. It has also been shown that transgenic expression of salmon delta-5 and delta-6 desaturase in zebrafish muscle inhibits *Vibrio alginolyticus* growth [32].

*V. vulnificus* causes serious, fulminant sepsis, mostly in patients with chronic liver diseases. After reaching the blood stream, the bacteria begin to proliferate and produce two main cytotoxins, VvhA and MARTXv_v_. VvhA, a haemolysin, is cytotoxic to host cells by causing necrosis or apoptosis depending on the amount of bacteria which cells exposure. MARTXv_v_ is required for *V. vulnificus* survival during infection by protecting itself from swallowing by phagocytes. Both cytotoxins build a microenvironment for *V. vulnificus* to facilitate bacterial dissemination and contribute to tissue damage [37]. To elucidate the anti-bacterial effect of n-3 PUFAs, we analyzed the immune and anti-inflammatory response of transgenic zebrafish in this study. We observed that transgenic fish were more resistant to the damaging effects of *V. vulnificus* infection, with repressed growth of *V. vulnificus* in the liver resulting in decreased mortality and reduced hepatocyte damage. Further, the bacterial contents in Wt liver were higher than in muscle (Fig. 5 (a)). It seems that the pathogen attacked the liver through the hepatic portal vein within a few hours after IP-injection. VvhA from *V. vulnificus* caused haemolysis and iron availability has been found to be related in pathogenic vibrios with haemolysin production [38]. In addition, liver is the storage depot of iron which is important to supply iron for accelerated erythropoiesis following a substantial loss of blood [39]. In clinical cases, patients with liver disease are more likely than healthy patients to die after *V. vulnificus* infection [40]. Therefore, we focused on liver and determined the expressions of inflammatory related genes.

NF-κB is activated by *V. vulnificus* infection [41]. The pro-inflammatory genes TNF-α and IL-1β are induced by the activation of NF-κB following LPS-induced infection [42]. However, n-3 PUFAs can directly bind PPAR-α to prevent the phosphorylation and translocation of NF-κB [43]. In agreement with this earlier finding, we describe here that NF-κB expression is suppressed in Fadsd6 and Elvol5a transgenic fish after *V. vulnificus* infection (Fig. 6). In addition, high n-3 PUFAs contents in transgenic fish reduced the expression of TNF-α and IL-1β to mitigate the spread of inflammation. Cox-2a, an inflammatory marker, is also expressed during many inflammatory responses induced by different pathogens [44]. In our results, the highest expression peaks of Cox-2a were at 3 and 6 h in Elvol5a and Fadsd6 transgenic fish, respectively; the expression peaks for transgenic fish occurred earlier than in Wt fish (9 h). As such, during the inflammatory process, the expression of Cox-2a in transgenic fish is rapidly increased to convert EPA to PGH_3_. Anti-inflammatory cytokines may be elevated to diminish the inflammatory response after *V. vulnificus* infection.

Moreover, IL-15 and lysozyme are secreted by macrophages and recruited to sites of infection to clear pathogens [45]. The expression levels of IL-15 and lysozyme were reduced in Wt fish after challenge with *V. vulnificus* as compared to transgenic fish. Expression of these factors can facilitate the removal of pathogen and increase the survival rate after infection. In addition, previous study demonstrated the potency of anti-bacteria of DHA is the highest than other long chain PUFAs [15]. With high contents of DHA in Fadsd6 transgenic fish (Fig. 2c); the expression levels of IL-15 and lysozyme were higher in Fadsd6 fish than Elvol5a fish. Our results may indicate high contents of DHA can enhance the expression level of IL-15 and lysozyme (Fig. 6e/f).

In summary, the transgenic fish described here can be used as an *in vivo* model for studying the bio-function of n-3 PUFAs, and for increasing EPA and DHA production in aquaculture fish to confer anti-bacterial and/or cold resistance. High n-3 PUFA content zebrafish may also provide a convenient platform with which to study the molecular mechanisms behind potentially lethal inflammation and septicemia due to *V. vulnificus* infection in human.

## Conclusion

In this study, liver-specific expression of salmon Fadsd6 or Elvol5a in transgenic fish was shown to enhance the bio-synthesis of n-3 PUFAs. Furthermore, transgenic fish exhibited resistance to *V. vulnificus* infection, with enhanced survival and anti-inflammatory effects. Moreover, these transgenic lines may serve as an *in vivo* model for studying the effects of n-3 PUFAs on bacterial infection and inflammation-associated diseases.

## Additional files

Additional file 1: Table S1. The quantitatiion of eicosapentaenoic acid (EPA), docosahexaenoic acid (DHA), docosapentaenoic acid (DPA) and total n-3 PUFA content in transgenic and Wt zebrafish. (DOCX 18 kb) Additional file 2: Figure S2. Quantitation of TUNEL signal analysis. The supplementary result is related to Fig. 4b. The TUNEL signals were counting. Values are presented as means ± SEM. Significance was determined by *T*-TEST (****P* < 0.001). (JPEG 259 kb) Additional file 3: Figure S1. Endogenous gene expressions of fatty acid synthesis genes were determined by real-time qPCR. The endogenous fatty acid synthesis genes, Fadsd2 (Fatty acid desaturase delta 2), Elovl2 (Elongase 2) and Elovl5 (Elongase 5) of zebrafish liver and muscle were analysis. (JPEG 301 kb)

## Abbreviations

n-3 PUFAs: Omega-3 polyunsaturated fatty acids
AA: Arachidonic acid (n-6, 20:4)
ALA: α-linolenic acid (n-3, 18:3)
DPA: Docosapentaenoic acid (n-3, 22:5)
DHA: Docosahexanoic acid (n-3, 22:6)
EPA: Eicosapentaenoic acid (n-3, 20:5)
Fadsd: Fatty acid desaturase
BF_3_: Boron trifluoride
TCBS: Thiosulfate-citrate-bile salts-sucrose
LPS: Popolysaccharide
*V. vulnificus*: *Vibrio vulnificus*
Cox-2a: Cyclooxygenase-2a
IL-1β: Interleukin-1 beta
NF-kB: Nuclear factor kappaB
TNF-α: Tumor necrosis factor-alpha
UPL: Universal probe library
