# Supplementary material for: Transgenic expression of omega-3 PUFA synthesis genes improves zebrafish survival during Vibrio vulnificus infection
Source: J Biomed Sci. 2015 Nov 17;22:103. doi: 10.1186/s12929-015-0208-1 (PMC4647518; doi:10.1186/s12929-015-0208-1)
Supplement: Additional file 1: Table S1. — The quantitatiion of eicosapentaenoic acid (EPA), docosahexaenoic acid (DHA), docosapentaenoic acid (DPA) and total n-3 PUFA content in transgenic and Wt zebrafish. (DOCX 18 kb) [file 12929_2015_208_MOESM1_ESM.docx]

| **Table S1** | **n-3 PUFA content in transgenic and Wt zebrafish** | | | |
| --- | --- | --- | --- | --- |
| **(mg/g)** | **EPA** | **DPA** | **DHA** | **Total n-3** |
| **Wt-contol** | **13.41±3.07** | **ND.** | **22.39±6.20** | **47.86±2.32** |
| **Wt-feed** | **14.59±0.89** | **ND.** | **19.61±1.43** | **39.94±2.18** |
| **Fadsd6** | **28.92±5.02*** | **7.80±1.62**** | **57.82±1.42**** | **100.89±10.83**** |
| **Elvol5a** | **30.16±6.58**** | **10.08±3.97**** | **48.21±11.93**** | **97.82±23.17**** |
| **n=5 * p< 0.05, ** p<0.01, ND. = non-detectable** | | | | |

**Supplementary table 1.** The quantitatiion of eicosapentaenoic acid (EPA), docosahexaenoic acid (DHA), docosapentaenoic acid (DPA) and total n-3 PUFA content in transgenic and Wt zebrafish.
