# Supplementary figures and images for: Transgenic expression of omega-3 PUFA synthesis genes improves zebrafish survival during Vibrio vulnificus infection
Source: J Biomed Sci. 2015 Nov 17;22:103. doi: 10.1186/s12929-015-0208-1 (PMC4647518; doi:10.1186/s12929-015-0208-1)

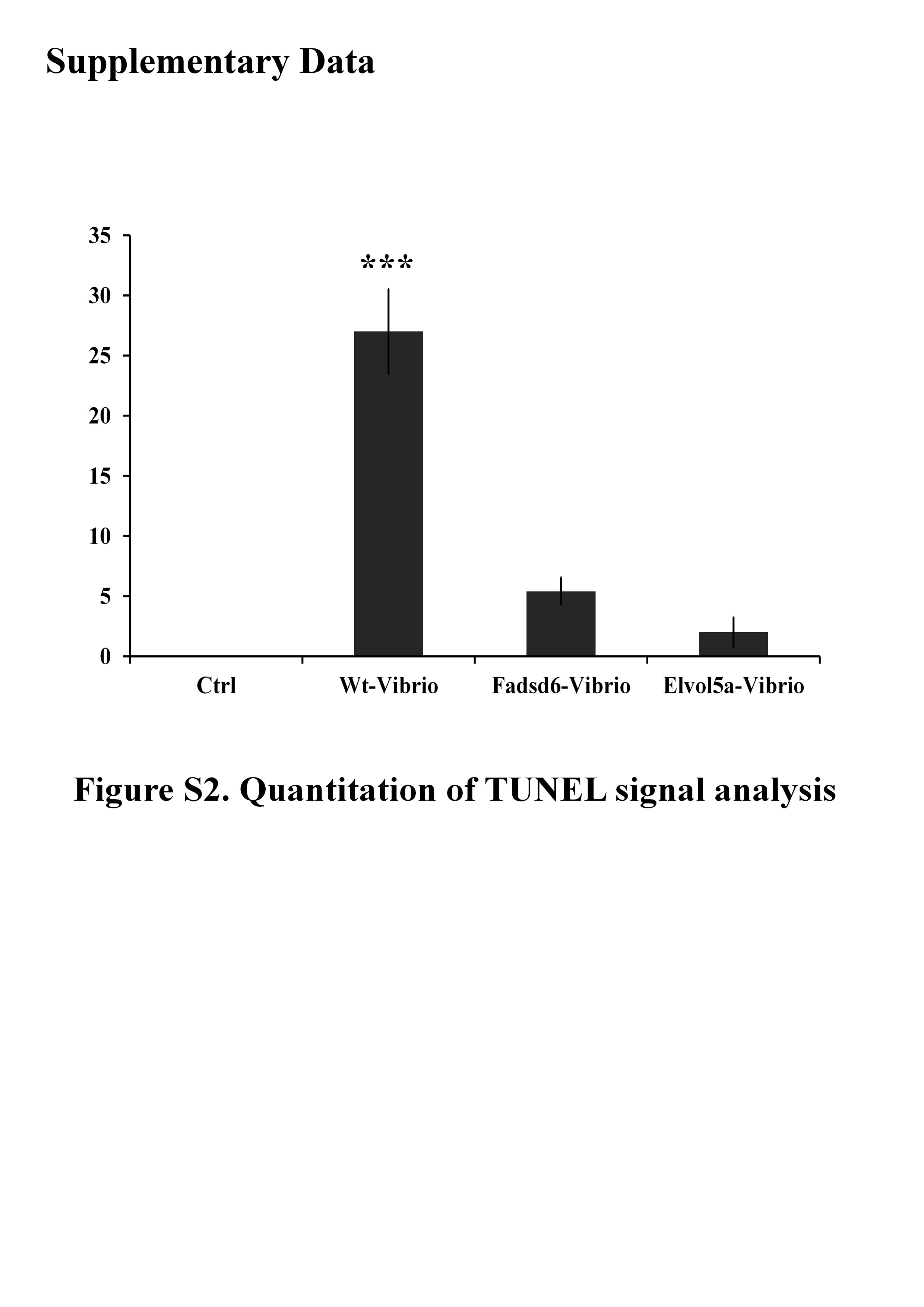

Supplement: Additional file 2: Figure S2. — Quantitation of TUNEL signal analysis. The supplementary result is related to Fig. 4b. The TUNEL signals were counting. Values are presented as means ± SEM. Significance was determined by T-TEST (***P < 0.001). (JPEG 259 kb) [file 12929_2015_208_MOESM2_ESM.jpg]

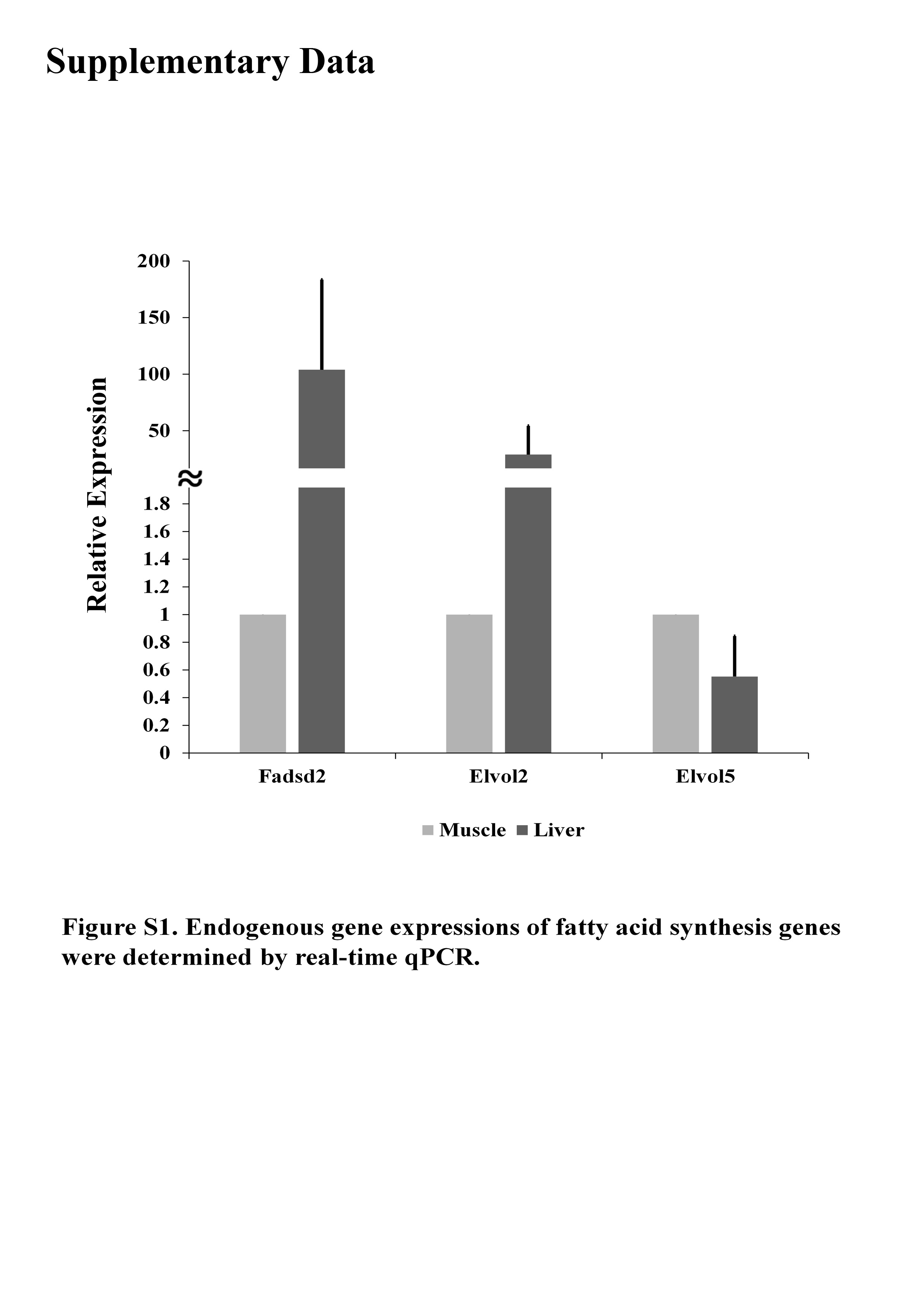

Supplement: Additional file 3: Figure S1. — Endogenous gene expressions of fatty acid synthesis genes were determined by real-time qPCR. The endogenous fatty acid synthesis genes, Fadsd2 (Fatty acid desaturase delta 2), Elovl2 (Elongase 2) and Elovl5 (Elongase 5) of zebrafish liver and muscle were analysis. (JPEG 301 kb) [file 12929_2015_208_MOESM3_ESM.jpg]
